# Integrated Lipidomic and Transcriptomic Analysis Reveals Lipid Metabolism in Foxtail Millet (*Setaria italica*)

**DOI:** 10.3389/fgene.2021.758003

**Published:** 2021-11-16

**Authors:** Haiying Zhang, Junyou Wang, Jing Zhao, Changqing Sun, Jin Wang, Qian Wang, Fei Qu, Xiaodong Yun, Zhiwei Feng

**Affiliations:** ^1^ College of Agriculture, Shanxi Agricultural University, Taigu, China; ^2^ Hebei Zhihai Technology Co., Ltd., Xingtai, China; ^3^ Shanxi Institute of Organic Dryland Farming, Shanxi Agricultural University, Taiyuan, China

**Keywords:** foxtail millet, UPLC-Q-TOF-MS/MS, lipidomics, transcriptome, association analysis

## Abstract

Foxtail millet (*Setaria italica*) as the main traditional crop in China, is rich in many kinds of high quality fatty acids (FAs). In this study, Ultra-high performance liquid chromatography-time-of-flight-tandem mass spectrometer (UHPLC-Q-TOF-MS/MS) was used to determine the lipids of JG35 and JG39. A total of 2,633 lipid molecules and 31 lipid subclasses were identified, mainly including thirteen kinds of glycerophospholipids (GP), eleven kinds of glycerolipids (GL), four kinds of sphingolipids (SP), two kinds of fatty acyls (FA) and one kind of sterol (ST). Among them JG35 had higher contents of diacylglycerols (DG) and ceramides (Cer), while triacylglycerols, phosphatidyl ethanolamine, phosphatidic acid, sterol, fatty acyls and pardiolipin (TG, PE, PA, ST, FA and CL) were higher in JG39. Meantime, the correlation analysis of lipidomics and transcriptomics was used to map the main differential lipid metabolism pathways of foxtail millet. The results shown that a differentially expressed genes (DEGs) of *FATA/B* for the synthesis of FA was highly expressed in JG35, and the related genes for the synthesis DG (*ACCase*, *KAS*, *HAD*, *KCS*, *LACS* and *GAPT*), TG (*DGAT* and *PDAT*) and CL (*CLS*) were highly expressed in JG39. The results of this study will provide a theoretical basis for the future study of lipidomics, improvement of lipid quality directionally and breeding of idiosyncratic quality varieties in foxtail millet.

## Introduction

Foxtail millet (*Setaria italica*) is one of the most ancient grain crops originating from China and is among the top five most important cereals worldwide (Qin et al., 2020; Zhao et al., 2021). The crop is drought and barren tolerant, with a short life cycle (Pan et al., 2018; Li et al., 2021). Dehusked foxtail millet is rich in many kinds of nutrients (Verma et al., 2015; Meherunnahar et al., 2018; Trivedi et al., 2018), and has a low-fat content (about 2.8–8.0%) with high-quality FAs. The main unsaturated fatty acids (UFAs) in dehusked foxtail millet are linoleic acid, oleic acid, and linolenic acid, whereas the main saturated fatty acids (SFAs) are palmitic acid, stearic acid, and arachidic acid. Among them, UFAs represent a large proportion, accounting for about 85.54% of the total FAs. UFAs exhibit unique biological activity, and thus, have significant physiological functions in the human body (Mccloy et al., 2004). Linoleic acid and linolenic acid are cell components and precursors of prostaglandins that participate in phospholipid synthesis. Also, they are closely related to cholesterol transport, which is crucial in regulating human physiological functions and safeguarding physical health (Fitzsimons et al., 2019; Samson et al., 2020; Saini and Rai, 2021).

Lipids are hydrophobic or bisexual organic molecules that are the main component of biofilm structures. Besides, they function as small signaling molecules and energy substances that mediate many biological processes. The International Commission on Lipopid Classification and Nomenclature classifies lipids into eight categories: fatty acyls (FA), glycerolipids (GL), glycerophospholipids (GP), sphingolipids (SP), saccharolipids (SL), sterol lipids (ST), prenol lipids (PR) and polyketides (PK) (Checa et al., 2015). Lipidomics is a systematic qualitative and quantitative analysis of various lipids within the body of an organism at the molecular level. It can be used to efficiently study the changes and functions of lipid families and lipid molecules in various biological processes, which is important in clarifying the processes and mechanisms of related biological activities (Wenk, 2005). Currently, the most common and convenient method for lipidomic analysis is high performance liquid chromatography-mass spectrometry (HPLC-MS/MS) and Ultra High Performance Liquid Chromatography-Time of Flight-Mass Spectrom (UHPLC-TOF MS/MS). The latter, as a high resolution mass spectrometry, has the characteristics of good stability, fast scanning speed and high sensitivity, and has been widely used in the extraction of a variety of chemical components (Wu et al., 2018; Singh et al., 2019; Yong et al., 2020).

Since its development, lipidomics has been widely applied in biomedicine, human health, and other aspects (Eggers et al., 2017; Kus et al., 2018; Wu et al., 2020; Ecker et al., 2021; Marasca et al., 2021). Lipids have been shown to play an essential role in plant growth, photosynthesis, and signal transduction (Hou et al., 2016; Rodríguez-López et al., 2017). Therefore, more and more studies on lipid metabolism have been conducted in plants. Yao et al. (2017) performed a comparative lipidomic analysis of lipid droplets in the mesocarp and seed tissues of Chinese tallow trees using LC-MS. The results showed that the most abundant triacylglycerol (TG) species in the mesocarp included one C18:1, two C16:0, and FAs. However, the three C18 FAs with higher unsaturated levels are dominant in the lipid droplets from grains. Non-targeted lipidomics has been conducted to study lipid antioxidation and galactose lipid remodeling in tomato plants under temperature stress (Spicher et al., 2016). In total, 791 lipid molecules were identified; and it was found that the remodeling of the thylakoid membrane in the chloroplast matrix is affected by fatty acid saturation in glycolipids and lipid oxidation levels at high temperatures.

With the completion of foxtail millet genome sequencing and the development of bioinformatics, it has provided convenient conditions for digging into the excellent functional genes of foxtail millet (Zhang et al., 2012). Transcriptomic sequencing can be used to sequence almost all transcripts in specific biological tissues or cells at a certain period. Additionally, it can be applied to study gene expression, structure, variable splicing and to predict new transcription (Patel et al., 2021; Sun et al., 2021; Wang et al., 2021; Zhou et al., 2021). Fei et al. (2020) employed transcriptome sequencing and RT-qPCR to determine the expression levels of 20 genes related to fatty acid synthesis in *Zanthoxylum bungeanum* seeds, the results of intergroup correlation and RDA analysis suggested that *ENR*, *ECR*, and *SAD1* are the key genes in fatty acid synthesis. Meng et al. (2021) used transcriptomics to predict the key genes of unsaturated fatty acid (UFA) synthesis and oil accumulation in *Paeonia lactiflora* seeds, including *MCAT*, *KASIII*, *FATA*, *SAD*, *FAD2*, *FAD3*, and *DGAT*, providing more comprehensive genomic resources for understanding the transcription of genes in *P. lactiflora* seeds.

Metabolomics considers organisms to be dynamic integration. It is applied to explore the process of metabolic changes caused by internal or external factors through statistical analysis (Rai et al., 2017). As a branch of metabolomics, lipidomics, in combination with transcriptology, can be used to identify and analyze the relevant genes in the metabolic pathway and precisely reflect the changes in the organism *per se* (Shen et al., 2016). However, no comprehensive analysis of lipid components and metabolism in foxtail millet has been reported. In this study, UHPLC-Q-TOF-MS/MS was used to compare and identify the lipid molecules of two different foxtail millet varieties. Further, transcriptomic analysis was used to explore the correlation between lipid metabolites and related gene expression patterns and identify the factors affecting lipid content in foxtail millet, which provides insights into the molecular biological mechanism of lipid metabolism, study of functional nutritional components and the selection of high quality foxtail millet varieties.

## Materials and Methods

### Plant Materials

Two foxtail millet varieties JG35 and JG39, were bred at the Institute of Foxtail Millet Crops, Hebei Academy of Agriculture and Forestry Sciences. Both of these two varieties were grown in summer sowing areas, and were resistant to the herbicide of imazethapyr and sethoxydim. However, they had greatly difference in the crude fat content. JG35 is a high-fat variety with the content of 5.4%, whereas JG39 is a low-fat variety, with the content of 2.9%. Samples were collected from the Yulin test site (109°21′ E, 37°56′ N, with an elevation of 1,120 m, annual average precipitation of 436 mm, and an annual average temperature of 8.0°C), seeds were selected 28 days after pollination with three replicates. And the transcriptome data of seeds were obtained from the NCBI Sequence Read Archive (SRA) under accessions with the SRR number 11840494, 11840495, 11840514, 11840501, 11840502, and 11840503, which were reported by Yuan et al. (2021).

### Sample Processing

Representative whole seeds without mildew, particles, or other impurities were selected and unshelled into foxtail millet using a JLGL-45 hulling mill (brand: Xinfeng, manufactured by Taizhou Grain Instruments Factory, Zhejiang Province). Next, the seeds were ground into powder using liquid nitrogen and mixed evenly for subsequent analysis. Precisely, 50 mg sample was weighed and put into an EP tube containing 200 μL water. The sample was then blended through whirl pooling for 30 s, then ground using friction bails for 12 min at 1,000 rpm. Next, the sample was subjected to 15 min of ultrasonic processing in an ice-water bath, then 480 μL extract (MTBE: methanol = 5:1) was added, followed by 30 s of blending through whirlpooling and 10 min of ultrasonic processing in an ice-water bath. After that, the sample was left to stand for 1 h at −20°C and centrifuged for 15 min at 4°C, 10,000 rpm. A total of 380 μL supernatant was carefully extracted and added into an EP tube. The extract was dried in a vacuum concentrator then mixed with 200 μL solution (dichloromethane: methanol = 1:1) for redissolution. Next, the mixture was blended through whirlpooling for 30 s and subjected to ultrasonic processing in an ice-water bath for 10 min. The sample was then centrifuged for 15 min at 4°C, 13,000 rpm, and 180 μL supernatant was carefully extracted and added into a vial. Exactly 10 μL of each sample was taken and mixed into the QC sample for testing on the machine. All reagents used were chromatographically pure.

### Chromatographic Condition

Chromatographic separation was performed on a Waters UPLC Acquity I-Class PLUS ultra-high performance liquid chromatography system. The chromatography column used was the Acquity UPLC CSH C18 (1.7 μM, 2.1 × 100 mm) column purchased from Waters. Positive ion mode: mobile phase A: 60% acetonitrile water solution, 10 mM ammonium acetate, 0.1% formic acid; mobile phase B: 90% isopropanol/acetonitrile solution, 10 mM ammonium acetate, 0.1% formic acid. Negative ion mode: mobile phase A: 60% acetonitrile water solution, 10 mM ammonium acetate, 0.1% formic acid; mobile phase B: 90% isopropanol/acetonitrile solution, 10 mM ammonium acetate, 0.1% formic acid. The gradient elution was programmed as follows: 0–2.0 min, 40% B; 2–18 min, 43%–100% B; 18–20 min, 40% B. Flow rate: 0.4 ml/min; injection volume: 5 μL; column temperature: 55°C. The sample was placed in a 10°C automatic sampler for analysis.

### Mass Spectrometry Conditions

Primary and secondary mass spectrometric data were collected under software (MassLynx V4.2, Waters) controlled MSe mode using a Waters Xevo G2-XS QTOF high-resolution mass spectrometer. Data on the low and high collision energy were collected by two channels in each data collection cycle. The low collision energy was 2 V, whereas the high collision energy interval was 10–40 V. The scanning frequency was 0.2 s/mass spectrum. ESI ion source parameters: capillary voltage: 2,000 V (positive ion mode) or −1,500 V (negative ion mode); taper hole voltage: 30 V; ion source temperature: 120°C; desolvent gas temperature: 550°C; blowback air flow rate: 50 L/h; desolvent gas flow rate: 900 L/h.

### Lipid Data Analysis

The original data collected by MassLynx V4.2 were put through peak recognition, peak extraction, peak alignment, and other data processing operations using Progenesis QI software. Identification was performed using Progenesis QI software’s online LMSD (Lipid Maps Structure Database) database and BMK’s self-built database, and theoretical fragment recognition was conducted. The Precursor Ion mass number deviation was within 100 ppm in each case, the fragment ion mass number deviation was within 50 ppm in each case (Want et al., 2010; Dunn et al., 2011; Kuhl et al., 2012). The extracted data were subjected to quantitative and lipid composition analyses, and diagram R language and SigmaPlot12.0 were drawn.

### Transcriptome Data Analysis

Differential expression analysis was performed using the DESeq2. The resulting *p* values were adjusted using the Benjamini and Hochberg’s approach for controlling the false discovery rate. Genes with an adjusted *p*-value < 0.01 and | log_2_FC | > 1 found by DESeq2 were assigned as differentially expressed. Gene Ontology (GO) enrichment analysis of the differentially expressed genes (DEGs) was implemented by the GOseq R packages based Wallenius non-central hyper-geometric distribution (Young et al., 2010), which can adjust for gene length bias in DEGs. And KOBAS (Mao et al., 2005) software was used to test the statistical enrichment of DEGs in KEGG pathways.

## Results

### Data Quality Assessment

#### Spectrum Comparison of the QC Sample

**FIGURE 1:**
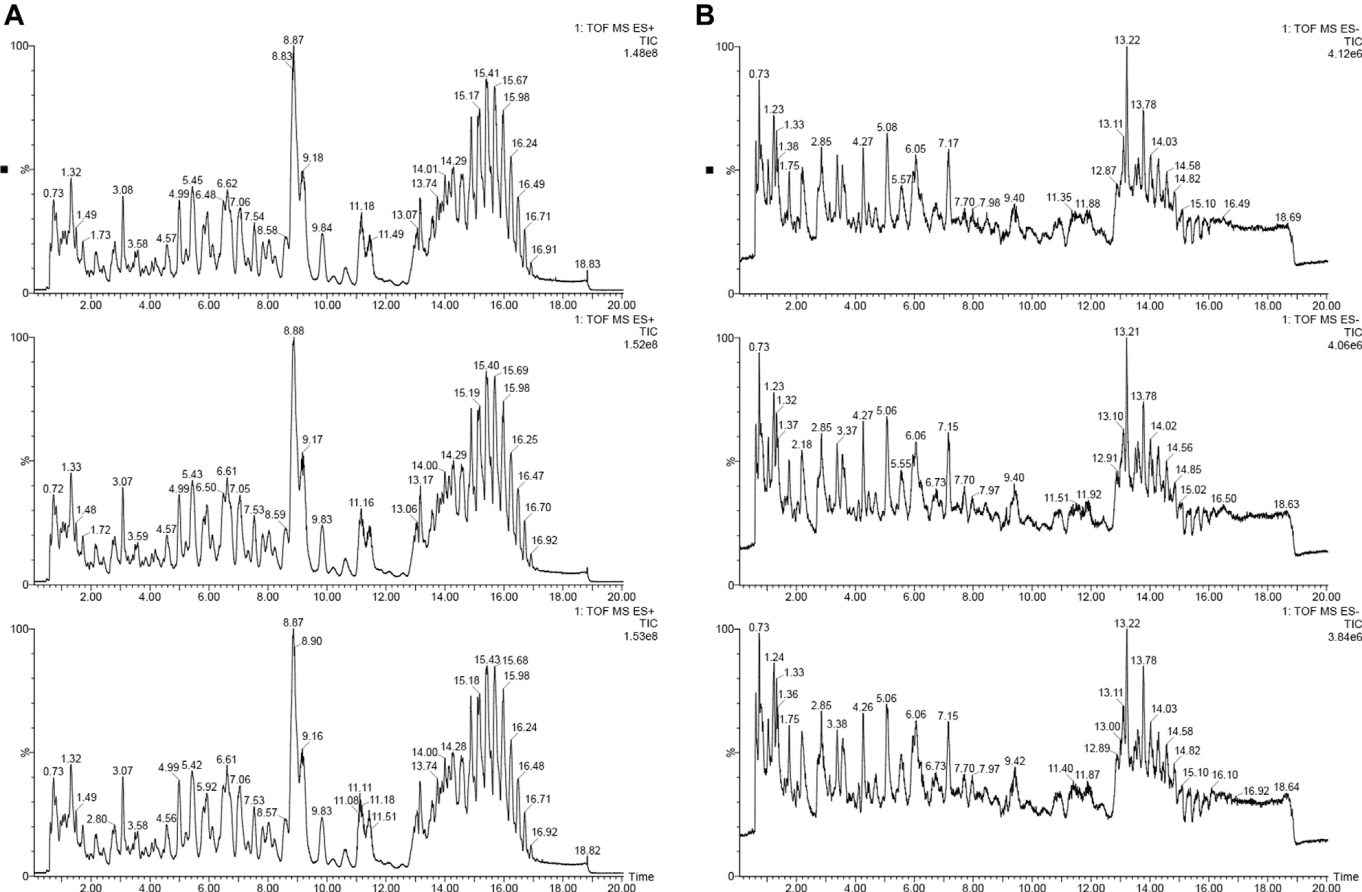
The base peak intensity chromatogram (BPI) of positive and negative ion [**(A)**: positive iron; **(B)**: negative ion].

In order to understand the sampling situation of the tested samples and the reliability of test results, the QC sample was added to monitor the stability and repeatability of the test system. As shown in Figure 1, the base peak chromatograms (BPI) of the three QC samples were overlapped and compared, and the results shown that the chromatographic peak response intensity and retention time of different samples overlapped, suggesting that the data collection system was stable and reliable enough for subsequent lipidomic analysis of foxtail millet.

#### Univariate Analysis of Lipid Molecules

**FIGURE 2:**
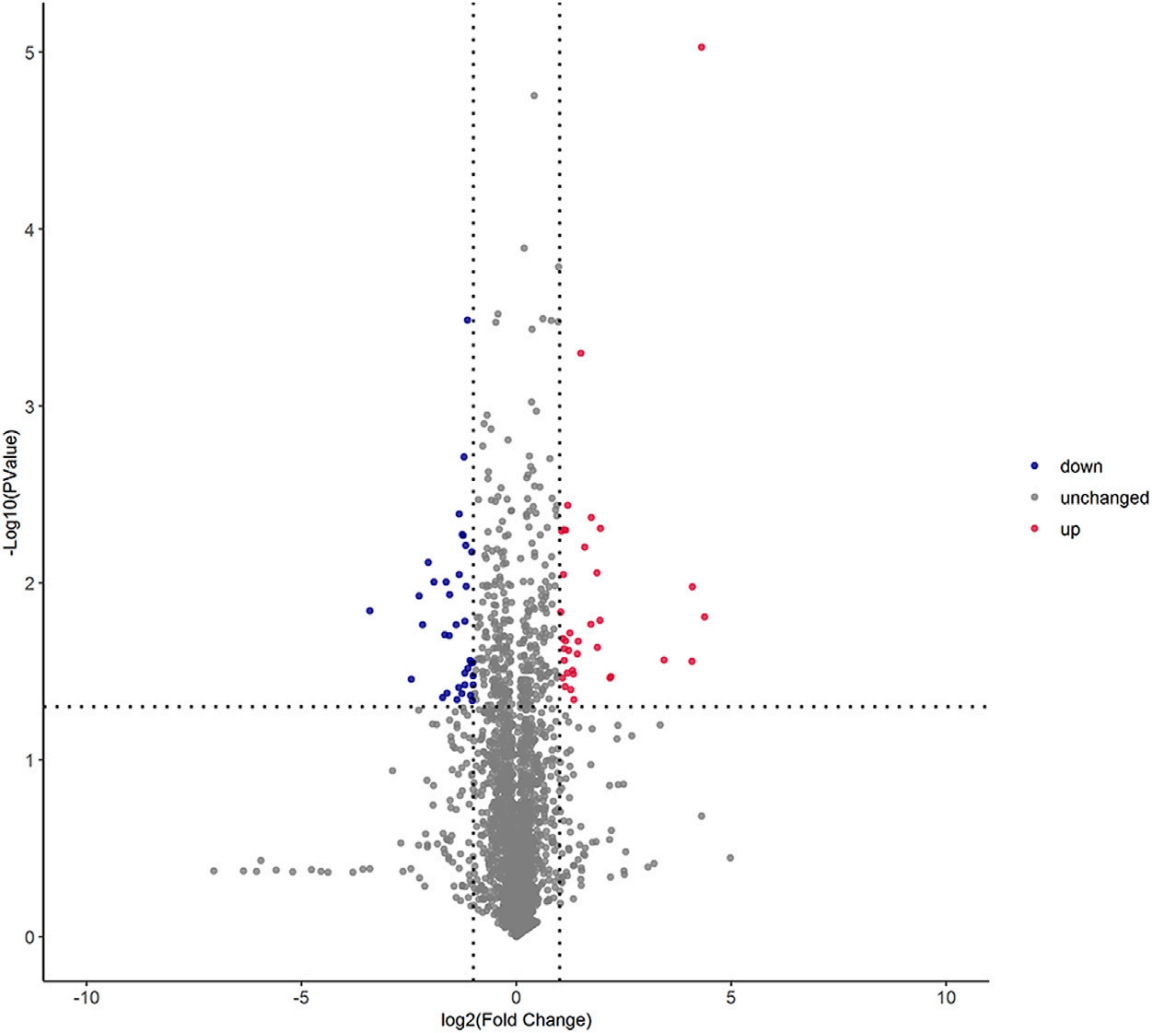
Volcano graph of differential lipid molecules in two foxtail millet. Note: Each dot in the volcano plot denotes one lipid. The x-axis denotes the fold change of each substance under comparison in the group (adopting logarithm with a base of 2), and the y-axis denotes the *p*-value of the *t*-test (adopting logarithm with a base of 10). The scatter size denotes the VIP value of the OPLS-DA model: the larger the scatter size, the greater the VIP value, and the more reliable the differentially expressed lipid obtained through screening. The blue spots denote lipids with down-regulated differential expression; the red spots denote lipids with up-regulated differential expression; the gray spots denote the detected lipids without significant differences.

The univariate statistical methods commonly used for differential analysis of two sample groups include variation fold analysis and the *t*-test/non-parametric test. All metabolites detected under positive and negative ion modes (including unidentified metabolites) were subjected to differential analysis based on univariate analysis, where the red denotes the differential metabolites with | log_2_FC | > 1, and adjusted *p-*value < 0.05. The volcano plot of differential expression was shown in Figure 2.

#### Multivariate Analysis of Lipid Molecules- Orthogonal Projections to Latent Structures-Discrimination Analysis

**FIGURE 3:**
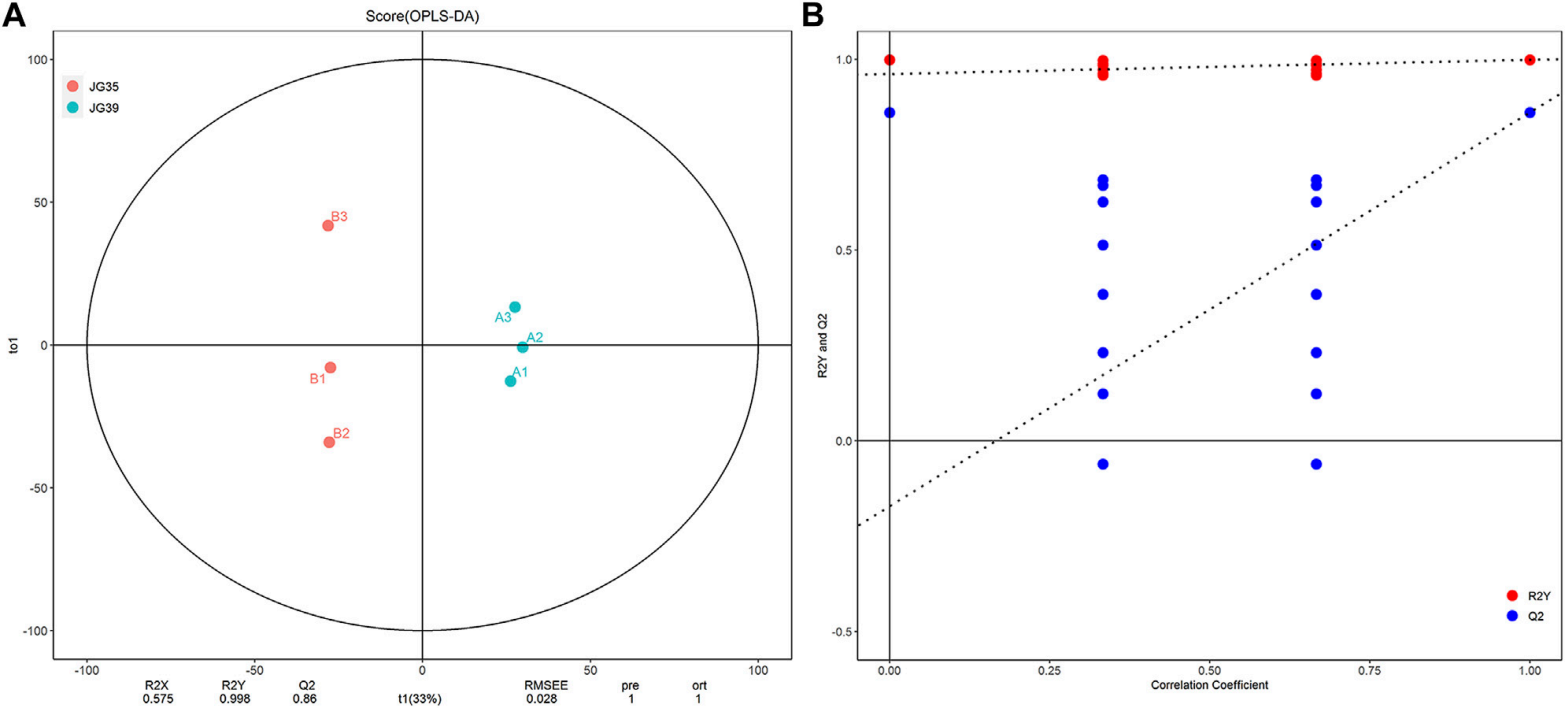
Multivariate statistical analysis of lipid molecules. **(A)** The Score chart of OPLS-DA. **(B)** Model validation diagram of OPLS-DA.

Orthogonal projection to latent structure-discriminant analysis (OPLS-DA) was performed to determine the lipid micromolecules with significant changes in the JG35 and JG39 millet varieties, thus building the relational model between the expression levels of lipids and the sample. The evaluation parameters of the model mainly included R^2^X, R^2^Y, and Q^2^. R^2^X and R^2^Y denote the explanatory powers of the model built for X and Y matrices, respectively, and Q^2^ denotes the prediction rate of the model. Generally, the closer the values of R^2^Y and Q^2^ to 1, the higher the stability and reliability of the model. Under normal circumstances, the model is considered effective when Q^2^ > 0.5, and excellent when Q^2^ > 0.9. According to our results, the parameters R^2^Y and Q^2^ of the model were 0.998 and 0.832, respectively (Figure 3A), indicating that the model was relatively reliable. To prevent overfitting of the model built, we performed permutation analysis to test the model and ensure its effectiveness. Normally, when the intercept between the Q^2^ regression line and the Y-axis is < 0.05, there is no overfitting of the model built. In this study, the intercept was uniformly <0.05 (Figure 3B), indicating that there was no over fitting of the model. Overall, these results suggest that the samples tested in this study have good repeatability and stability, thus can be used for subsequent lipid metabolism analysis.

### Lipid Composition Analysis

#### Lipid Composition Identification and Analysis in Grains

**FIGURE 4:**
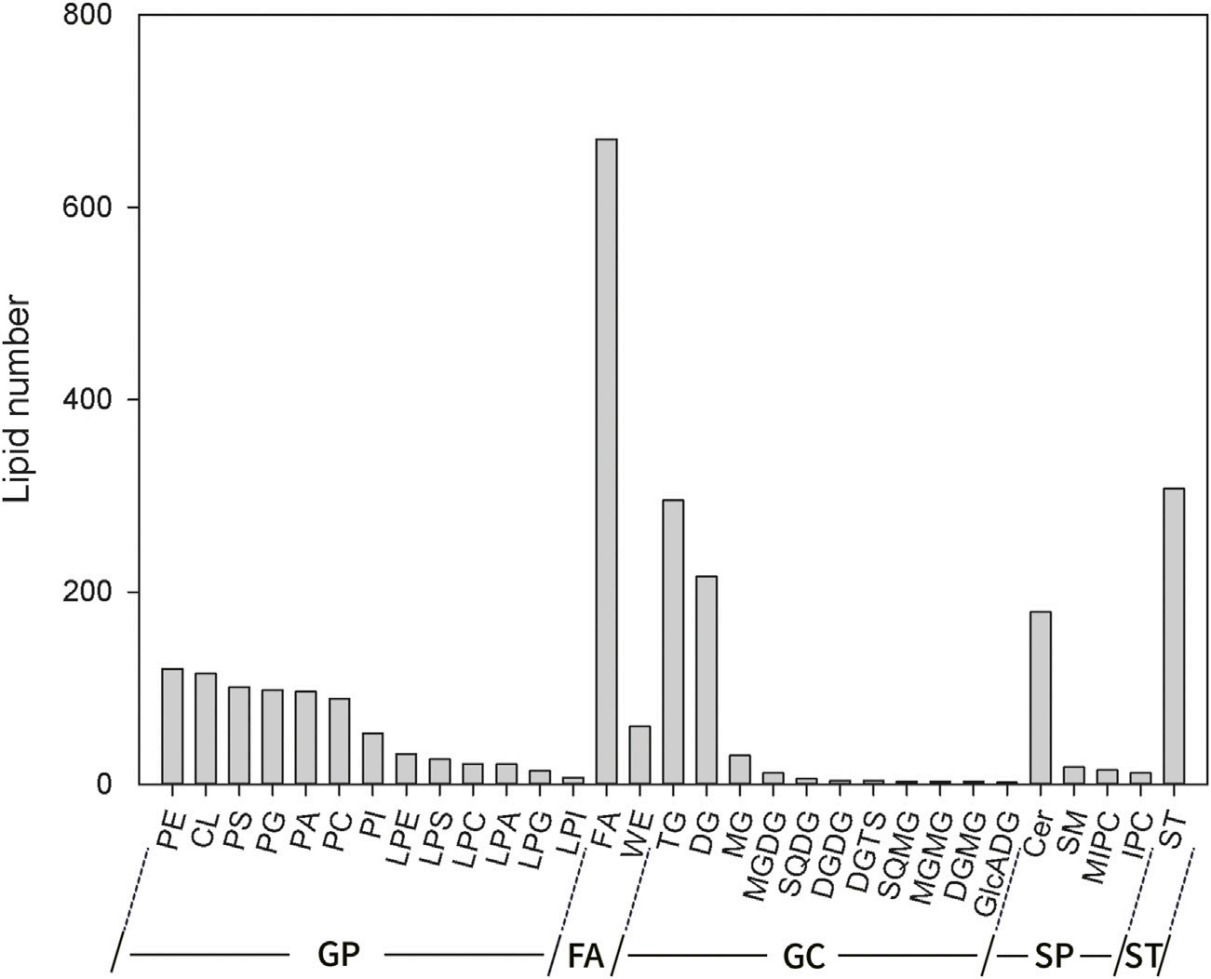
Lipid composition of foxtail millet.

Complete lipidomic analysis was performed on grain samples from two foxtail millet varieties using UHPLC-QTOF-MS. According to the results, 31 lipid subclasses (2,633 lipid molecules) were identified, including 13 kinds of GP, 11 kinds of GL, 4 kinds of SP, 2 kinds of FA and 1 kind of ST (Figure 4). Among them, GP had the largest number (793) including 120 for PE, 115 for CL, 101 for PS, 98 for PG, 96 for PA, 89 for PC, 53 for PI, 32 for LPE, 26 for LPS, 21 for LPC, 21 for LPA, 14 for LPG, 7 for LPI. The followed in succession was two kinds of FA included FA (671) and WE (60). 578 kinds of GL species included 295 for TG, 216 for DG, 30 for MG, 12 for MGDG, 6 for SQDG, 4 for DGDG, 4 for DGTS, 3 for SQMD, 3 for MGMG, 2 for DGMG, 2 for and GlcADG. 224 kinds of SP included 179 for Cer, 18 for SM, 15 for MIPC, 12 for IPC. The last category included 307 kinds of ST. It can be seen that the number of lipid molecules varied significantly across different lipid subclasses, and FA had the largest number.

#### Lipid Content of Grains

**FIGURE 5:**
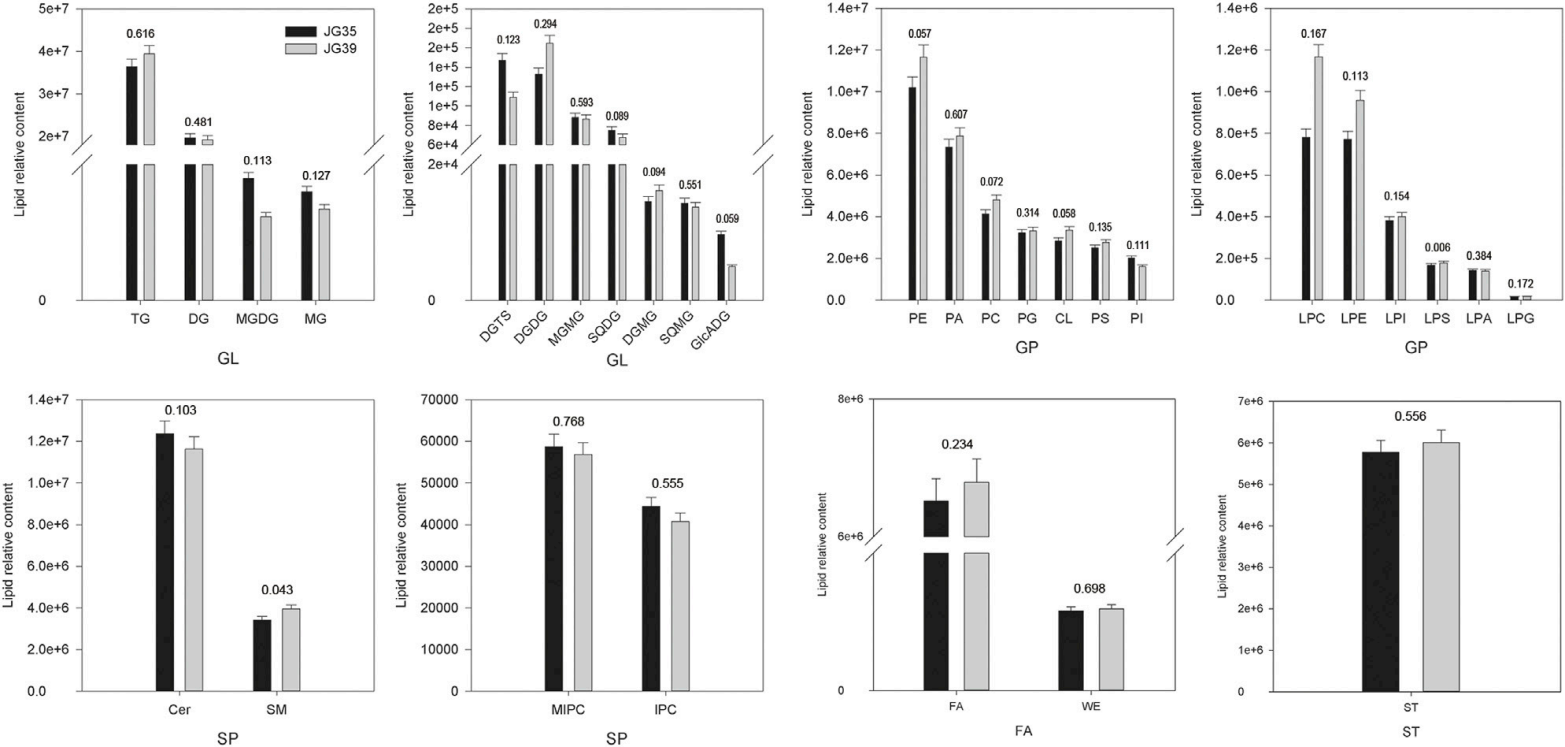
Comparison of lipid subgroup contents between JG 35 and JG39 (The values in the figure represent *p* values).

Among the two foxtail millet varieties, JG35 had a relative lipid content of 1.21×10^8^, whereas the content was 1.27×10^8^ in JG39. Overall, the relative contents of various lipid compositions in JG35 and JG39 were similar, and GL content was the highest, accounting for 48.24 and 47.55% of the total lipid content, respectively (Figure 5). Specifically, the contents of TG and DG were relatively higher in the two foxtail millet varieties, and the relative content were 3.64 × 10^7^vs3.94 × 10^7^, and 1.97 × 10^7^vs1.92 × 10^7^, respectively. The next content was GP, accounting for 28.44 and 30.01% of the total lipid content, respectively. SP (containing Cer, SM, MIPC and IPC) accounted for 13.10 and 12.31%, respectively. FA (including FA and WE) accounted for 5.46 and 5.42%, respectively. The contents of ST in JG35 and JG39 were 5.77 × 10^6^ and 6.01 × 10^6^, respectively. In conclusion, it was found that JG35 had higher DG and Cer, while JG39 had higher TG, PE, PA, ST, FA, and CL.

### Differential Analysis of Lipid Molecules

The number of lipid molecules in JG35 and JG39 were compared to determine their lipid differences. A total of 72 significantly differential lipid molecules (| log_2_FC | >1 and adjusted *p-*value < 0.05) were selected from the 2,633 lipid molecules identified through qualification (Supplementary Table S1). Particularly, the numbers of differential lipids were 11 for FA, 9 for Cer, 6 for CL, 6 for ST, 4 for DG, 4 for PA, 4 for TG, 3 for PC, 3 for PE, 3 for PS, 2 for PI, 2 for IPC, 2 for SM, 2 for LPE, 2 for MG, 1 for SQDG, 1 for LPC, 1 for LPG, 1 for MIPC, 1 for LPS, 1 for LPA, 1 for PG, 1 for WE, and 1 for MGDG. The 11 FAs with the most difference were FA (22:5), FA (21:2), FA (30:2), FA (13:8), FA (19:0), FA (24:0), FA (6:2), FA (21:1), FA (14:4), FA (18:0) and FA (20:4), which most of them were unsaturated FA.

Through comparative analysis, it was found that GP had the most differential lipids, including 3 kinds of PS, 2 kinds of PI, 1 kind of PG, 3 kinds of PE, 3 kinds of PC, 4 kinds of PA, 1 kind of LPS, 1 kind of LPG, 2 kinds of LPE, 1 kind of LPC, 1 kind of LPGA and 6 kinds of CL. JG35 had higher contents of PS 35:1, 35:4, PI 21:1, PE DO-34:4, PE 22:2; O2, PC O-31:0, PC 23:2; O2, PA 36:4, PA O-28:0, LPE O-27:1; O, LPC 18:1, CL 57:3, CL 72:7, CL 76:1, CL 76:5; and JG39 had higher contains of PS 39:0, PI 35:1, PE O-34:4, PC 40:1, PA 34:1, PA 38:2, LPS O-28:1; O, LPG 17:0, LPE O-21:0; O, LPA 18:0, CL 68:4, CL 78:9. MG O-18:1, O-16:0; O, DG 40:6, DG 32:1, TG 45:0, SQDG 32:1, MGDG 34:4; O were higher in JG35, and DG 31:1, dO-34:4, TG 59:9, 64:8, 62:16 were higher in JG39. JG35 had higher contents of FA 22:5, 21:2, 19:0, 24:0, 21:1, 14:4, WE 11:2, JG39 had higher contents of FA 30:2, 13:8, 6:2, 18:0, 20:4. For the subclasses of SP, JG35 had higher contents of Cer 41:1; O3, 42:1; O4, 34:2; O3, 38:0; O4, SM 42:0; O2, IPC 36:0; O3, JG39 had higher contents of Cer 34:1; O2, 33:1; O2, 32:3; O3, 39:1; O3, 36:1; O2, SM 42:2; O2, IPC 46:0; O3, MIPC 44:0; O2. For ST, ST 31:1; O, ST 27:1; O4, ST 27:1; O3, ST 27:1; O4; S were higher in JG35, and ST 27:3; O, 27:5; O5 were higher in JG39.

**FIGURE 6:**
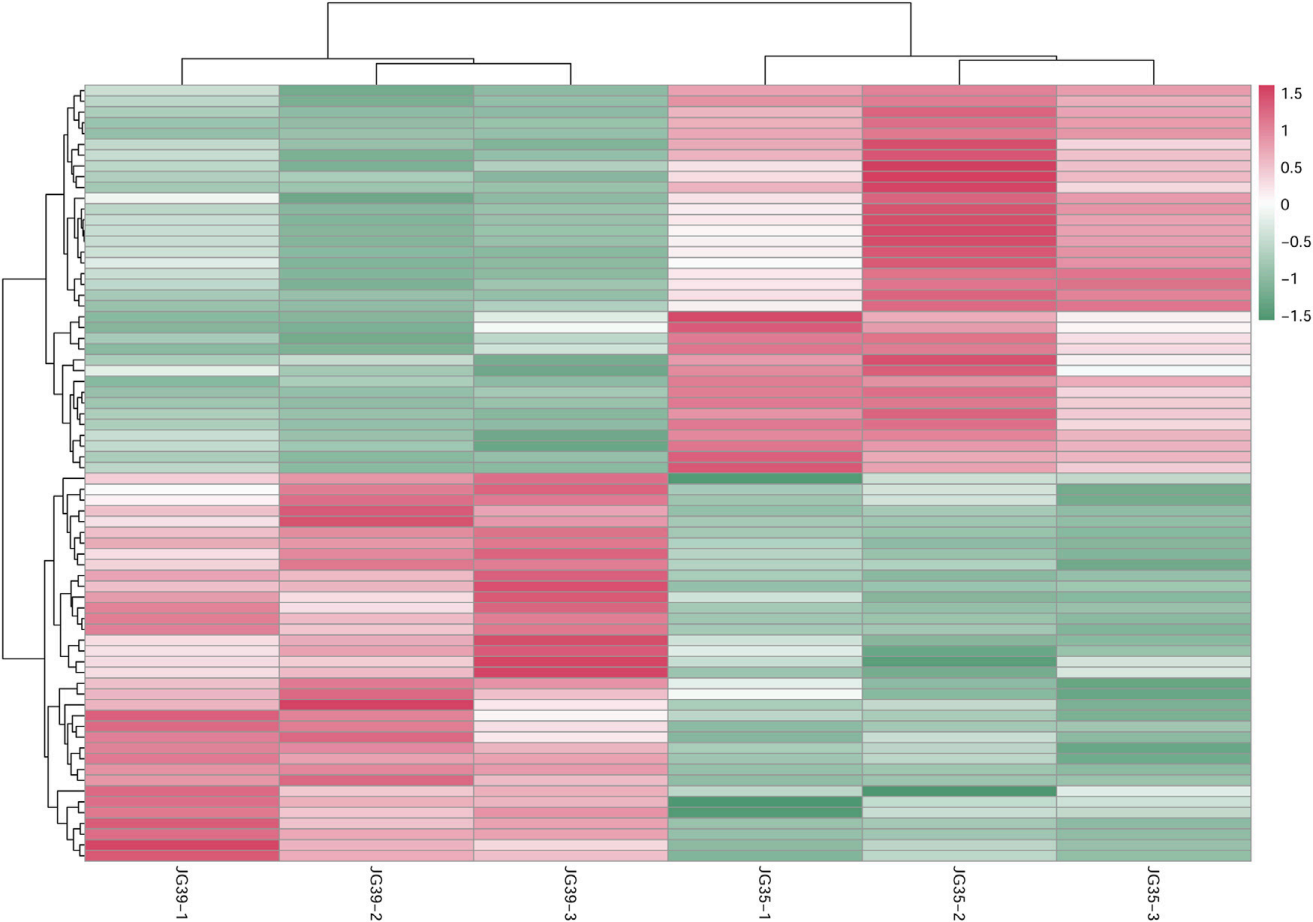
Cluster diagram of differential lipid metabolism between JG35 and JG39.

The expression levels of qualified, significantly differential lipids were adopted for the hierarchical clustering of samples in different groups (Figure 6). This was done to evaluate the significance of differential lipid screening and comprehensively and visually display the relationships between samples and the different expression modes of lipids in different samples. According to the results, the three replicates of various samples clustered together. These differential lipid metabolites all presented similar accumulation modes in the repetitions of various samples, suggesting that they had similar reaction steps in the metabolic process. Moreover, these lipids showed obvious metabolite differences in various samples, demonstrating the significance and representativeness of the screened lipid metabolites.

### Analysis of Differentially Expressed Genes

By comparing the gene expression of JG35 and JG39, 1165 DEGs were found based on | log_2_FC | > 1 and FDR<0.01. Among them, 383 genes were up-regulated in JG35 and 782 genes were up-regulated in JG39 (Supplementary Table S2). Through GO enrichment, 736 genes of these differential genes were enriched into 48 different functional classifications (Supplementary Figure S1). In the biological processes, these differential genes are mainly concentrated in metabolic process, cellular process, single-organism process, etc. In the cell components, these genes are mainly concentrated in cells, cell part, organelle, membranes, membrane part, etc. In molecular function classification, these differential genes are mainly concentrated in binding, catalytic activity, transporter activity and structural molecule activity. In order to further understand the functional roles of these DEGs in the pathway, KEGG pathway enrichment analysis was conducted, and the results showed that DEGs were significantly enriched in Ribosome and Oxidative phosphorylation pathways. Furthermore, some of the DEGs were enriched in fatty acid elongation, steroid biosynthesis, fatty acid degradation and linoleic acid metabolism (Supplementary Figure S2), which provided a molecular basis for the further study on lipid-related genes.

### Combined Analysis of Transcriptomic and Lipidomic

**FIGURE 7:**
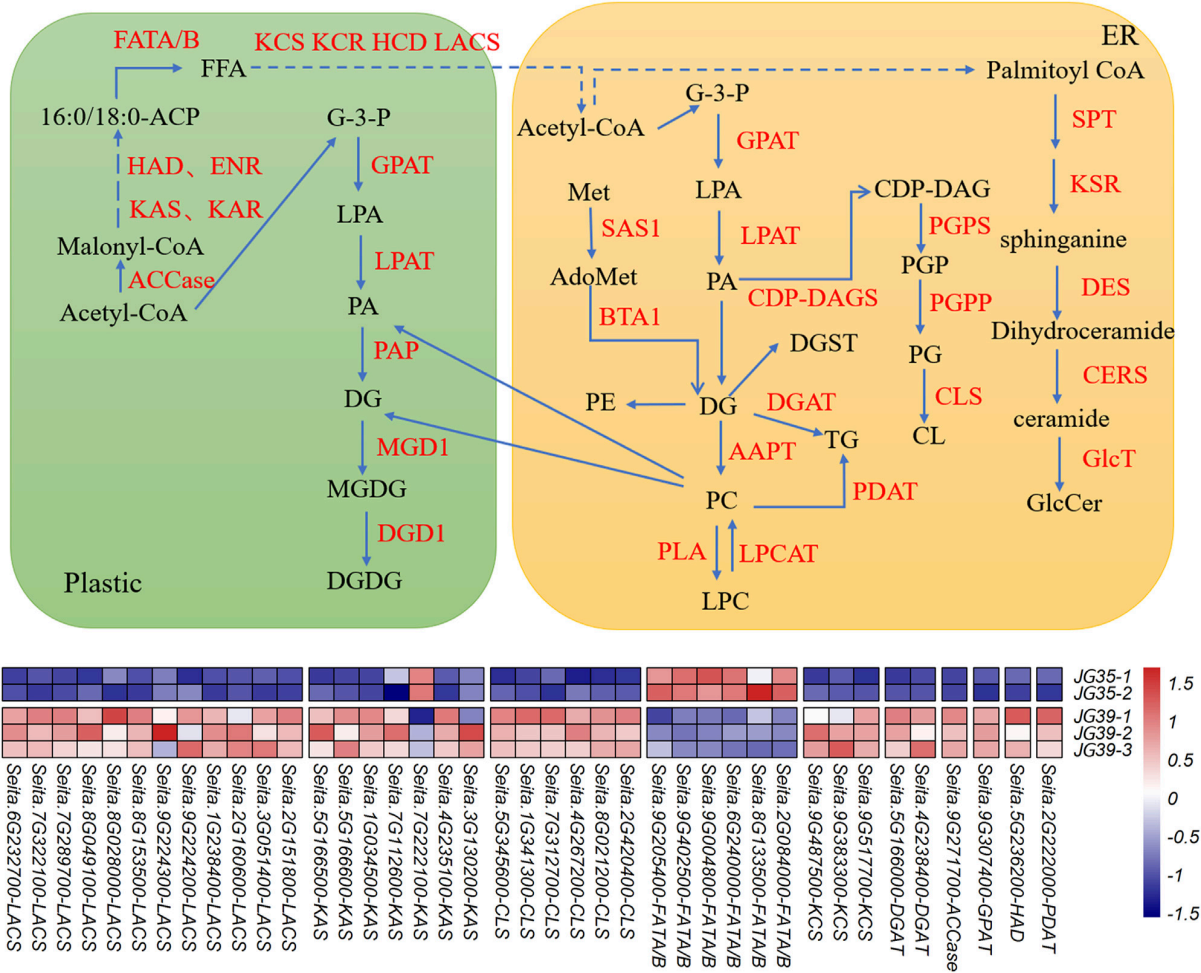
Lipid metabolism pathway map of foxtail millet. Note: AAPT, aminoalcoholphosphotransferase; ACCase, acetyl-CoA carboxylase; Acetyl CoA, acetyl-coenzyme A; AdoMet, S-adenosylmethionine; BTA1, betaine lipid synthase; CDP-DAG, CDP-dia-cylglycerol; CDP-DAGS, CDP-DAG synthase; CERS, ceramide synthase; CL, cardiolipin; CLS, cardiolipin synthase; DES, dihydroceramide desaturase; DGD1, digalactosyldiacylglycerol synthase 1; DGAT, diacylglycerol acyltransferase; ECR, trans-2,3-enoyl-CoA reductase; ENR, enoyl-ACP-reductase; FATA/B, acyl-ACP thioesterase A/B; FFA, free fatty acids; GlcT: glycosyltransfferases; G-3-P, glycerol-3-phosphate; GPAT, glycerol-3-phosphate acyltransferase; HAD, hydroxyacyl-ACP dehydratase; HCD, 3-hydroxacyl-CoA dehydratase; KAR, β-ketoacyl-ACP reductase; KAS, ketoacyl-ACP synthase; KCR, 3-ketoacyl-CoA reductase; KCS, 3-ketoacyl-CoA synthase; KSR, 3-ketosphinganine reductase; LACS, long-chain acyl-CoA synthetase; LPAT, lysophosphatidic acid acyltransferase; LPCAT, lysophosphatidyl choline acyltransferase; MeT, methionine; MGD1, monogalactosyldiacylglycerol synthase 1; PAP, phosphatidic acid phosphatase; PLA, phospholipase A; PDAT, phospholipid:diacylglycerol acyltransferase; PG, phosphatidylglycerol; PGP, phosphatidylglycerol phosphate; PGPP, PGP phosphatase; PGPS, phosphatidylglycerophosphate synthase; SAS1, S-adenosylmethionine synthetase; SPT, serine palmitoyltransferase.

The transcriptome and lipidome data of JG35 and JG39 were integrated. Genes related to lipid metabolism in the transcriptome were screened out according to the functional annotation of DEGs (Supplementary Table S3), and the major lipid molecules in the lipid group were combined for association analysis. Meanwhile, the molecular regulatory network of the main lipid metabolism in foxtail millet was constructed (Figure 7). However, according to transcriptomic analysis, JG35-1 did not show any significant difference in terms of correlation with other samples within the group or samples in the other group (Supplementary Figure S3). Thus, the sample was not selected for preparing the heat map of differential genes.

PA and DG are both important intermediate products in the metabolic process of lipids. Their *de novo* synthesis starts with G-3-P (3-phospho-glycerol) and FAs as initial substrates. Denovo synthesis occurs in both endoplasmic reticulum (ER) and plasmid pathways. Acetyl coenzyme A serves as the primer in the synthesis of FAs. malonyl-ACP provides a dicarbon unit for each step of the extension reaction. Malonyl thioester first engages in condensation reactions with acetyl-CoA and then goes through acceptor reactions with acyl-ACP. These reactions are catalyzed by KAS condensing enzymes, resulting in the formation of carbon-carbon bonds. It takes three different KAS condensing enzymes to generate one 18C fatty acid, containing KAS І, KAS ІІ, and KAS ІІІ. In the condensation reaction process, the extension of the fatty acyl group chain further requires the participation of KAR, ENR, and HAD. The long-chain acyl groups are hydrolyzed by FATB, generating 18:0-ACP. 18:0-ACP is further hydroxylated under the action of FATA/B. Finally, FAs are activated into acyl coenzyme A *via* LACS, which is exported into the ER.

The UHPLC-QTOF-MS analysis showed that among the differential lipids, JG35 has a higher FA content than JG39, and the expression levels of synthesis-related genes *FATA/B* were also higher in JG35. For DG, they were exhibited a relatively high content in JG39, and the expression level of *ACCase*, *KAS*, *KCS*, *LACS* and *GPAT* corresponding to DG synthesis were also high in the transcriptomes. Similarly, JG39 had higher TG content, and the corresponding synthase genes *DGAT* and *PDAT* expression were upregulated. Besides, CL was higher in JG39 and the expression of *CLS* was also higher (Supplementary Figure S2). Genes related to differential lipid metabolite molecules with relatively low contents were not detected in the transcriptomes, possibly because of insufficient detection depth. However, further studies are needed to determine the precise causes.

## Discussions

### Lipidomic Comparison of Two Different Foxtail Millet

In this study, the lipidomics of two different foxtail millet varieties, JG35 and JG39, were explored using the HPLC-QTOF-MS method. As a result, 31 lipid subclass and 2,633 lipid molecule were identified. By comparison, it was found that JD35 had higher DG and Cer, while TG, PE, PA, ST, FAs and CL were found to be relatively higher in JD39. DG is a structured lipid formed by glycerate after a hydroxyl replaces one fatty acid. It is deemed as a safe and healthy edible lipid, foxtail millet varieties with a high DG content have essential functions in reducing visceral fat and blood fat and inhibiting weight increase (Wan et al., 2020). As a decomposition product of sphingomyelin in biofilm bilayers, Cer is a universally recognized second messenger that plays an extensive and vital role in the growth, proliferation, differentiation, apoptosis, and damage of cells (Shikata et al., 2003). For TG, its level is closely related to the risk of cardiovascular disease with arteriosclerosis, the effective control of TG is of great significance to reduce the risk of cardiovascular system and reduce the incidence, death and disability of cardiovascular diseases (Spitler and Davies, 2020; Raposeiras-Roubin et al., 2021). GP, as a kind of phospholipid with a large content in the body, is a component of biofilm and one of the active substances on the surface of cell membrane. Meanwhile, GP participates in the recognition and signal transduction of protein by cell membrane (Chung et al., 1995; Grant and Guest, 2012). FA, the raw material for other compounds, can esterify cholesterol, lower blood cholesterol and triglycerides, improve brain cell activity and improve memory and thinking (Morton et al., 2020; Ulug and Nergiz-Unal, 2021). STs are widely found in roots, stems, leaves, fruits and seeds of plants, which plays a certain role in inhibiting tumors, promoting metabolism and regulate hormone levels (Marttinen et al., 2012; Sadek et al., 2017; Shimada et al., 2021).

### Combined Analysis of Lipidome and Transcriptome

Many studies on the combination of lipid metabolism and transcriptomics have been reported, Zhang et al. (2021) combined bicolor epidermal lipidomics and transcriptomics to reveal the function of cuticular wax and cutin in maintaining drought resistance in sorghum. Also, Liang et al. (2019) applied transcriptomics and lipidomics to analyze the genes related to neutral lipid accumulation in *Nannochloropsis* under nitrogen deficiency/sufficient conditions. In this study, the composition and metabolism of lipids were comprehensively analyzed in two different foxtail millet varieties, it was found that JG35 had higher content of FA than JG39, and the expression of *FATA/B*, a key gene for the synthesis of FA (Aznar-Moreno et al., 2016; Ma et al., 2018; Correa-Aguado et al., 2021), was also increased. However, JG39 had higher levels of DG, as the key or rate-limiting enzyme in the *de novo* synthesis, the expression of *ACCase* gene was higher in JG39. And the expression levels of *KAS*, *HAD*, *KCS*, and *LACS* were also upregulated in the synthetic process from plastic to ER for acetyl-CoA in JG39. Meanwhile, the expression of *GPAT*, a related synthesis gene was also increased. As the key genes for the biosynthesis of TG (Woodfield et al., 2018), *DGAT* and *PDAT* were upregulated in JG39, and the content of TG was higher. PA can form CDP-dia-cylglycerol (CDP-DAG) by the catalysis of CDP-DAG synthase (CDP-DAGS) and then produce CL by the catalysis of cardiolipin synthase (CLS) (Zhang et al., 2020). Here, JG39 had higher CL and *CLS* was upregulated. Feng et al. (2016) identified 10 *KAS* genes involved in fatty acid synthesis in eucommia ulmoides seeds by transcriptome sequencing. Bao et al. (2021) found that fatty acid biosynthesis related genes *LACS6, DGD1*, *ACAT1*, *AGPAT*, *WSD1*, *EGY2*, *and oleosin* were highly expressed during late development of *Prunus Pedunculata* Pall seeds. Xiu et al. (2018) found key genes in oil biosynthesis of *Paenpnia*, containing *ACCase*, *LPCAT*, *FADs*, *DGAT.* These reveals the possible reason for the high contents of differential lipids for DG, TG and CL.

China has abundant foxtail millet variety resources; however, there is limited research on the effective utilization and mining of different resources. Also, many excellent resources remain unexplored, limiting the breeding of special foxtail millet varieties. The study of investigating the composition and change in foxtail millet by lipidomics is of great significance for the development of functional foods, the utilization of germplasm resources and the breeding of special varieties of foxtail millet.

## Conclusion

In this paper, UHPLC-Q-TOF-MS system was firstly used to compare the lipid content and composition of two different foxtail millet varieties containing JG35 and JG39, and combined with transcriptome data to construct the molecular regulatory network of major lipid metabolism preliminatively in foxtail millet. A total of 2,633 kinds of lipid molecules and 31 kinds of lipid subclasses were identified by lipid detection. By comparing, the lipid composition was not significantly different. However, there were differences in the contents of each subcategory, JG35 had higher contents of DG and Cer, and the contents of TG, PE, PA, ST, FA, and CL were higher in JG39. By the comparison of differential lipid, it was found that the key genes *FATA/B* for the synthesis of FAs was highly expressed in JG35, and the related genes (*ACCase*, *KAS*, *HAD*, *KCS*, *LACS*, and *GAPT*) for the synthesis of DG were highly expressed in JG39. Besides, in JG39 the expression of *DGAT* and *PDAT* for the synthesis of TG was also higher, and *CLS* related to CL synthesis was upregulated. The two varieties contain different contents of lipids which are beneficial to human health, so the development of functional foods and the breeding of new varieties can be carried out selectively according to different needs.

## Data Availability

The raw data supporting the conclusion of this article will be made available by the authors, without undue reservation.

## Glossary

Cer: ceramides
CL: cardiolipin
DG: diacylglycerols
DGDG: digalactosyl-diacylglycerol
DGMG: digalactosylmonoacylglycerol
DGTS: diacylglyceryltrimethylhomo-Ser
FA: fatty acyls
FC: fold change
GlcADG: glucuronosyldiacylglycerol
IPC: inositolphosphoryl-ceramides
LPA: lysophosphatidic-acid
LPC: lyso-posphatidyl choline
LPE: lyso-posphatidyl ethanolamine
LPG: lyso-phosphatidylglycerol
LPI: lyso-phosphatidyl inositol
LPS: lipopolysaccharide
MIPC: mannosyl-inositolphosphoryl-ceramides
MG: monoacylglycerols
MGDG: monogalactosyl-diacylglycerol
MGMG: monogalactosylmonoacylglycerol
PA: phosphatidic acid
PC: phosphatidyl choline
PE: phosphatidyl ethanolamine
PG: phosphatidylglycerol
PI: phosphatidyl inositol
PIP: phosphatidylinositol diphosphate
PS: phosphatidyl serine
SM: sphingomyelin
SQDG: sulfoquinovosyl-diacylglycerol
SQMG: sulfoquinovosylmonoacylglycerols
ST: sterol
TG: Triacylglycerols
WE: wax esters.
