# Supplementary figures and images for: Integrated Lipidomic and Transcriptomic Analysis Reveals Lipid Metabolism in Foxtail Millet (Setaria italica)
Source: Front Genet. 2021 Nov 16;12:758003. doi: 10.3389/fgene.2021.758003 (PMC8635157; doi:10.3389/fgene.2021.758003)

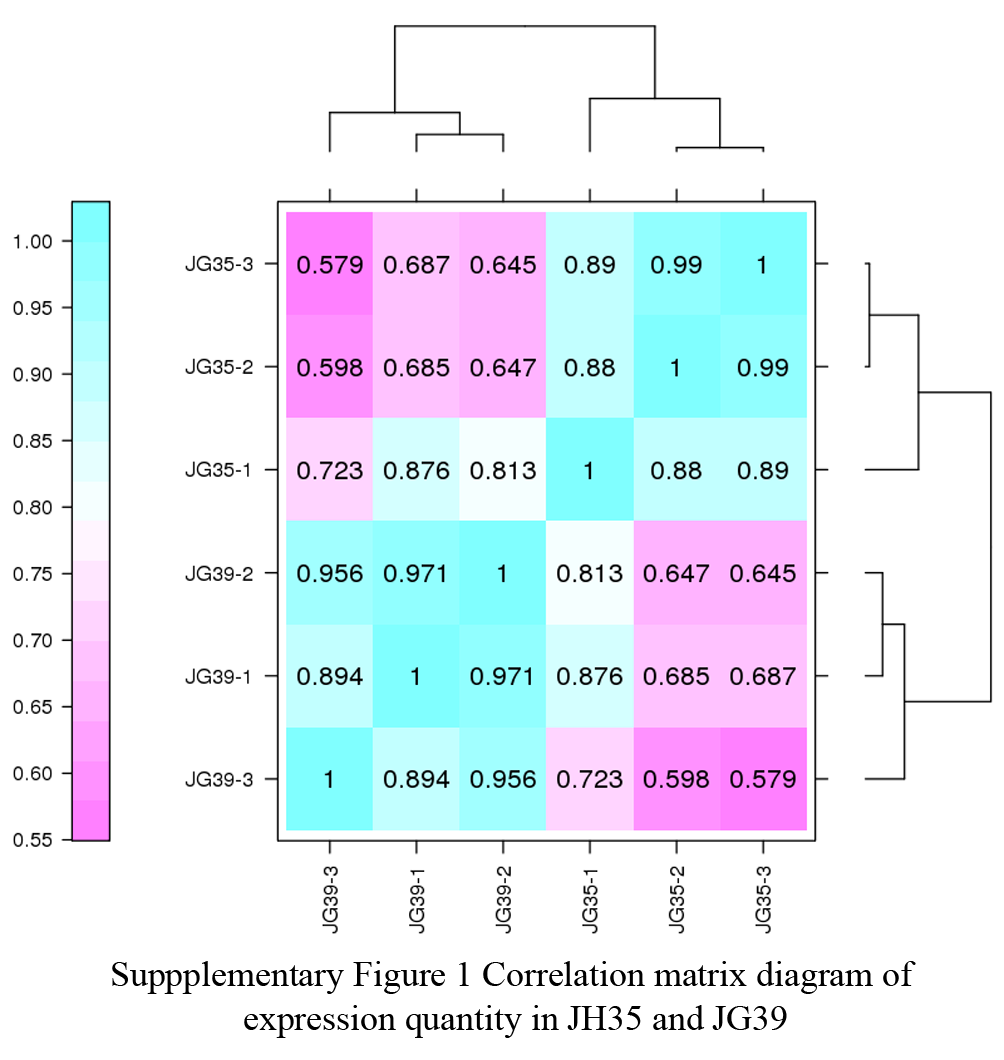

Supplement: Supplementary file 3 [file Image3.TIF]

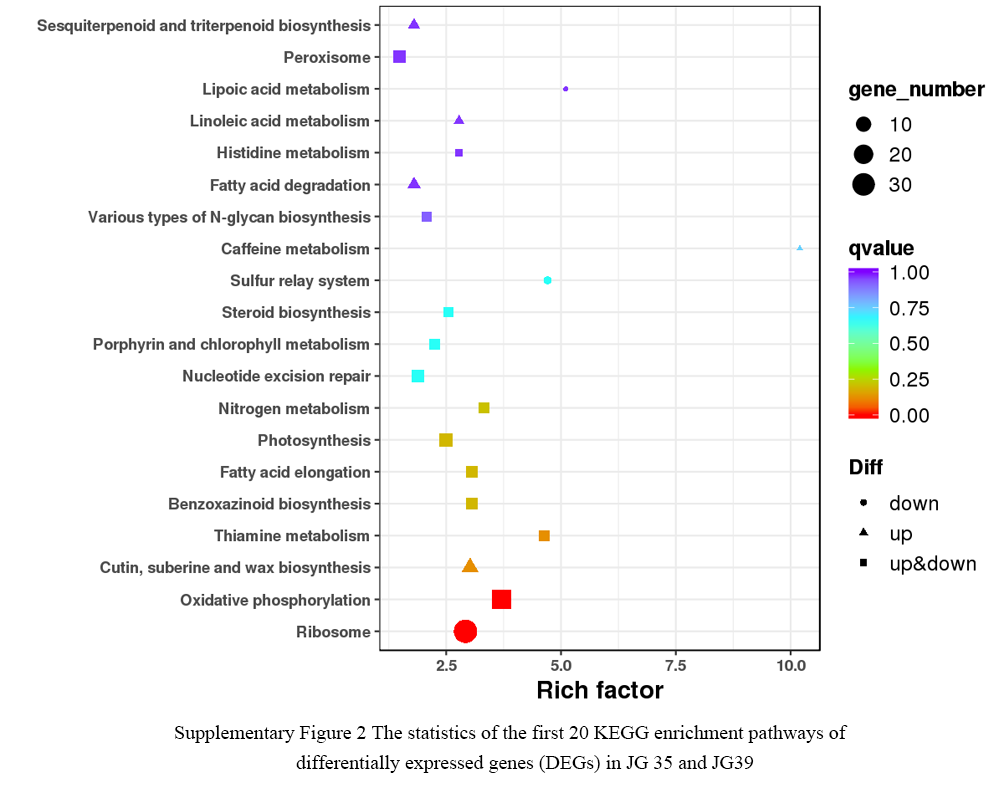

Supplement: Supplementary file 4 [file Image2.TIF]

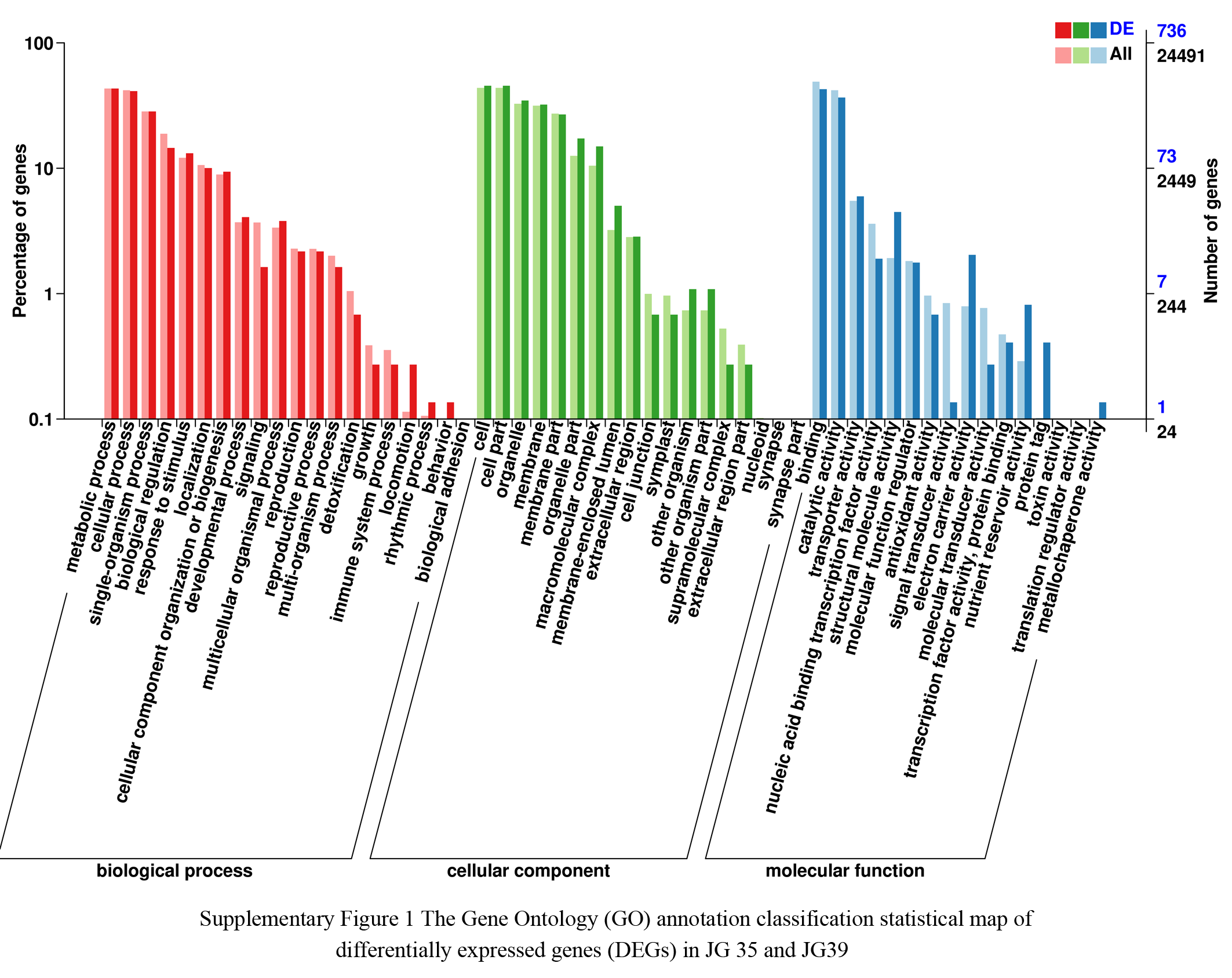

Supplement: Supplementary file 5 [file Image1.TIF]
